# Golimumab-Induced Anti-NuMA-1 (Nuclear Mitotic Apparatus Protein 1) Antibodies in a Rheumatoid Arthritis Patient: A Case Report

**DOI:** 10.7759/cureus.72918

**Published:** 2024-11-03

**Authors:** Imane Bensaghir, Latifa Tahiri, Yassine Taoufik, Hanan Rkain, Fadoua Allali

**Affiliations:** 1 Department of Rheumatology B, Ayachi Hospital, Ibn Sina Hospital Center, Faculty of Medicine and Pharmacy, Mohammed V University, Rabat, MAR; 2 Immunology, Immcell Laboratory, Rabat, MAR; 3 Department of Exercise Physiology and Autonomous Nervous System, Faculty of Medicine and Pharmacy, Mohammed V University, Rabat, MAR

**Keywords:** antinuclear antibodies, biotherapy, case report, dmards, golimumab, lupus-like symptoms, numa 1 antibodies, rheumatoid arthritis, tnf-alpha inhibitors

## Abstract

Anti-nuclear mitotic apparatus (NuMA) 1 antibodies are uncommonly detected in routine antinuclear antibody (ANA) screening. We present the case of a 65-year-old female with rheumatoid arthritis undergoing golimumab biotherapy who developed lupus-like symptoms including photosensitivity, fatigue, weakness, myalgias, alopecia, oral ulcers, and worsening of arthritis. Elevated serum levels of NuMA-1 antibodies were detected using indirect immunofluorescence (IIF) on HEp-2 cells with a titer of 1:1000, but no other ANA patterns were associated. The patient's symptoms were successfully treated with oral prednisone, leading to complete resolution.

This case highlights a potential association between golimumab and the induction of NuMA-1 antibodies, a previously unreported phenomenon, emphasizing the importance of vigilant monitoring for patients receiving golimumab, especially those with risk factors for autoimmune disorders, to early detect lupus-like symptoms and prevent complications.

## Introduction

Anti-nuclear mitotic apparatus (NuMA) antibodies 1 and 2 are autoantibodies that target the mitotic spindle apparatus (MSA), a component of the nucleus. These antibodies were first identified in 1981 as a subtype of antinuclear antibodies (ANA) [1]. These NuMA proteins, with a molecular weight of 238 kDa, are found in the nucleus during interphase and migrate to the spindle poles during cell division [2,3]. They play a role in microtubule movement and spindle stability [4]. Five distinct NuMA antigens have been identified: NuMA1, NuMA2, centrosome (CE), middle body (MB), and F-centromere (CENP-F) [5]. Anti-NuMA1 and anti-NuMA2 antibodies are often detected in the blood of individuals with autoimmune diseases. In some cases, these antibodies are found alongside other antibodies targeting specific antigens. Consequently, anti-NuMA patterns have been suggested as potential markers for autoimmune conditions [6]. However, anti-NuMA antibodies are rarely found in routine ANA screenings of human blood. These antibodies have been observed in individuals with a variety of connective tissue diseases, organ-specific autoimmune conditions, overlap syndromes, infections, and cancers, but their clinical importance is not fully understood [7-9].

We describe the first case of detected NuMA-1 antibodies in a female patient with rheumatoid arthritis (RA) undergoing golimumab biotherapy.

## Case presentation

A 65-year-old woman with a history of well-controlled asthma and osteoporosis, treated with inhaled corticosteroids and alendronate, respectively, has been under follow-up for RA since 2011. During this time, she was treated with several disease-modifying antirheumatic drugs (DMARDs), including methotrexate, sulfasalazine, and leflunomide, but discontinued each due to allergic skin rashes.

After receiving subcutaneous injections of etanercept 50 mg weekly for a year, which was discontinued due to loss of efficacy, she underwent intravenous infliximab infusions at a dose of 3 mg/kg at weeks 0, 2, 6, and then every eight weeks for two years. While she experienced clinical improvement, infliximab was discontinued due to side effects, including dyspnea and chest tightness, which appeared during infusions but quickly subsided after administration of 120 mg of solumedrol.

Subsequently, she received tocilizumab infusions for three months at a dose of 8 mg/kg per month, demonstrating a good clinical response. However, tocilizumab was discontinued due to stock shortages related to the COVID-19 pandemic. She then began golimumab subcutaneous injections at a dose of 50 mg per month, which were discontinued four months later due to the development of lupus-like symptoms, including photosensitivity, fatigue, weakness, myalgias, alopecia, oral ulcers, and worsening of arthritis (Table 1).

**Table 1:** Different DMARDs received by the patient and reason for discontinuation. DMARDs, disease-modifying antirheumatic drugs; cDMARDs, conventional disease-modifying antirheumatic drugs; bDMARDs, biological disease-modifying antirheumatic drugs

DMARDs	Reason for discontinuation
cDMARDs	
Methotrexate	Skin rash
Sulfasalazine	Skin rash
Leflunomide	Skin rash
bDMARDs	
Etanercept	Loss of efficacy
Infliximab	Allergic reaction (dyspnea and tightness of chest)
Tocilizumab	Stock shortage due to COVID-19 pandemic
Golimumab	Lupus-like symptoms: photosensitivity, fatigue, weakness, myalgias, alopecia, oral ulcers, and worsening of arthritis

**Figure 1:**
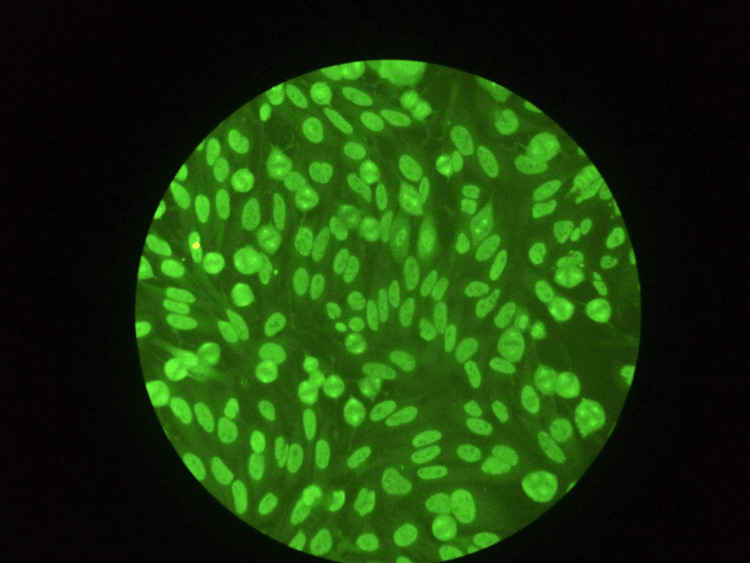
NuMA-1 antibodies pattern detected by IIF on HEp-2 cells. IIF, indirect immunofluorescence; NuMA, nuclear mitotic apparatus

The patient's symptoms prompted hospitalization and a comprehensive blood panel. All tests were normal except for ANA, detected by indirect immunofluorescence (IIF) on HEp-2 cells. A NuMA-1 fluoroscopic pattern was observed with a titer of 1:1000 (Figure 1), but no other ANA patterns were associated. Additional antibody tests, including anti-double-stranded DNA (dsDNA), antibodies against extractable nuclear antigens (ENA), anti-thyroglobulin (TG), anti-thyroperoxidase (TPO), anti-myeloperoxidase (MPO), and anti-proteinase 3 (PR-3), were negative. Serum cryoglobulins over four weeks were also negative.

The patient's symptoms were managed with oral prednisone at a dose of 0.5 mg/kg/day for a week, followed by tapering over a week and discontinuation. As a result, the patient's symptoms fully resolved and improved. Additionally, a pharmacovigilance report was filed for this reaction with a Vigibase number of 11-309-451-317.

## Discussion

While there are antibodies targeting the AMA that may not be easily identified, the pattern of ANA and their specificity make them valuable markers in assessing rheumatic diseases. Despite the fact that antibodies directed against NuMA are uncommon, their occurrence and clinical significance have been previously described in the literature [8].

The reported prevalence of anti-NuMA antibodies varies across different studies. Bonaci-Nikolic et al. found a low frequency of anti-MA antibodies (0.9%) in a five-year study involving 6,270 patients [8]. Moreover, Fritzler et al. detected anti-NuMA-1 antibodies in 0.16% of healthy female blood donors using HEp-2 cells [10]. In patients with autoimmune symptoms, anti-NuMA-1 antibodies were found in 0.36% of cases [8]. Furthermore, Auer-Grumbach and Achleitner identified anti-NuMA-1 antibodies in 0.23% of screened pathological sera [11].

A recent retrospective cohort study of 73,079 serum samples collected between 2013 and 2018 found that anti-NuMA antibodies are primarily associated with primary Sjogren's syndrome and systemic lupus erythematosus (SLE). These antibodies may be helpful in diagnosing these conditions when other autoantibodies are not detected. Additionally, individuals with primary Sjogren's syndrome or SLE who also have anti-NuMA antibodies tend to have less severe clinical symptoms and laboratory findings, suggesting that NuMA antibodies may be a positive prognostic marker [12].

The case we presented involves a 65-year-old female patient with RA who was treated with a TNF-alpha inhibitor, golimumab. The lupus-like symptoms she experienced following treatment initiation are well-established side effects of this drug, as documented in the literature. Indeed, the induction of lupus autoimmunity by anti-TNF agents has been previously observed in phase II and III clinical trials [13]. Systematic monitoring for autoimmunity in inflammatory rheumatism patients receiving anti-TNF therapy reported rates of 20-60% for ANA, 15-20% for anti-DNA, and 15-20% for anti-histones [14-18]. However, to date, there have been no documented cases of NuMA-1 antibody induction.

NuMA-1 antibodies have been implicated in autoimmune diseases such as SLE, and while our patient did not have a prior serum dosage of ANA or NuMA-1 antibodies, the development of lupus-like symptoms following the initiation of golimumab biotherapy indicates a potential association with the high serum levels of NuMA-1 antibodies. This association suggests that the antibodies were induced by golimumab biotherapy.

While cases of drug-induced lupus by TNF-alpha inhibitors have been extensively documented, this report represents the first instance of NuMA-1 antibody induction.

## Conclusions

This case report presents a unique instance of a patient with RA undergoing golimumab therapy who developed lupus-like symptoms associated with elevated levels of anti-NuMA-1 antibodies. These antibodies were detected by IIF on HEp-2 cells, the NuMA-1 fluoroscopic pattern was observed with a titer of 1:1000, but no other ANA patterns were associated. The findings highlight the importance of vigilant monitoring for patients receiving golimumab, especially those with a history of autoimmune disorders or other risk factors. Early detection of lupus-like symptoms can enable prompt intervention, potentially preventing severe complications and improving patient outcomes.

## References

[1] McCarty GA, Valencia DW, Fritzler MJ, Barada FA (1981). A unique antinuclear antibody staining only the mitotic-spindle apparatus. N Engl J Med.

[2] Compton DA, Szilak I, Cleveland DW (1992). Primary structure of NuMA, an intranuclear protein that defines a novel pathway for segregation of proteins at mitosis. J Cell Biol.

[3] Zeng C (2000). NuMA: a nuclear protein involved in mitotic centrosome function. Microsc Res Tech.

[4] Kisurina-Evgenieva O, Mack G, Du Q, Macara I, Khodjakov A, Compton DA (2004). Multiple mechanisms regulate NuMA dynamics at spindle poles. J Cell Sci.

[5] Bradwell AR, Hughes RG, Harden EL (2003). Atlas of HEp-2 Patterns and Laboratory Techniques.

[6] Maria Elena Soto ME, Becerril NH, Ríos GR (2019). Clinical association of antinuclear antibodies (ANA) anti-NuMA1 and anti- NuMA2 (anti-HsEg5) in Patients with autoimmune and cardiovascular disease. J Clin Chem Lab Med.

[7] Grypiotis P, Ruffatti A, Tonello M (2002). Significato clinico dei quadri fluoroscopici specifici per il fuso mitotico in pazienti affetti da malattie reumatiche. Reumatismo.

[8] Bonaci-Nikolic B, Andrejevic S, Bukilica M, Urosevic I, Nikolic M (2006). Autoantibodies to mitotic apparatus: association with other autoantibodies and their clinical significance. J Clin Immunol.

[9] Mozo L, Gutiérrez C, Gómez J (2008). Antibodies to mitotic spindle apparatus: clinical significance of NuMA and HsEg5 autoantibodies. J Clin Immunol.

[10] Fritzler MJ, Pauls JD, Kinsella TD, Bowen TJ (1985). Antinuclear, anticytoplasmic, and anti-Sjogren's syndrome antigen A (SS-A/Ro) antibodies in female blood donors. Clin Immunol Immunopathol.

[11] Auer-Grumbach P, Achleitner B (1994). Epidemiology and clinical associations of NuMA (nuclear mitotic apparatus protein) autoantibodies. J Rheumatol.

[12] Arcani R, Bertin D, Bardin N (2021). Anti-NuMA antibodies: clinical associations and significance in patients with primary Sjögren's syndrome or systemic lupus erythematosus. Rheumatology (Oxford).

[13] Pisetsky DS (2000). Tumor necrosis factor alpha blockers and the induction of anti-DNA autoantibodies. Arthritis Rheum.

[14] Charles PJ, Smeenk RJ, De Jong J, Feldmann M, Maini RN (2000). Assessment of antibodies to double-stranded DNA induced in rheumatoid arthritis patients following treatment with infliximab, a monoclonal antibody to tumor necrosis factor alpha: findings in open-label and randomized placebo-controlled trials. Arthritis Rheum.

[15] Atzeni F, Ardizzone S, Sarzi-Puttini P (2005). Autoantibody profile during short-term infliximab treatment for Crohn's disease: a prospective cohort study. Aliment Pharmacol Ther.

[16] Vermeire S, Noman M, Van Assche G (2003). Autoimmunity associated with anti-tumor necrosis factor alpha treatment in Crohn's disease: a prospective cohort study. Gastroenterology.

[17] Allanore Y, Sellam J, Batteux F, Job Deslandre C, Weill B, Kahan A (2004). Induction of autoantibodies in refractory rheumatoid arthritis treated by infliximab. Clin Exp Rheumatol.

[18] De Rycke L, Kruithof E, Van Damme N (2003). Antinuclear antibodies following infliximab treatment in patients with rheumatoid arthritis or spondylarthropathy. Arthritis Rheum.

